# *Arabidopsis* TNL-WRKY domain receptor RRS1 contributes to temperature-conditioned RPS4 auto-immunity

**DOI:** 10.3389/fpls.2013.00403

**Published:** 2013-10-17

**Authors:** Katharina Heidrich, Kenichi Tsuda, Servane Blanvillain-Baufumé, Lennart Wirthmueller, Jaqueline Bautor, Jane E. Parker

**Affiliations:** Department of Plant-Microbe Interactions, Max-Planck Institute for Plant Breeding ResearchCologne, Germany

**Keywords:** *resistance* gene pair, temperature shift, EDS1 signaling, biotic stress, programed cell death, transcriptional reprograming

## Abstract

In plant effector-triggered immunity (ETI), intracellular nucleotide binding-leucine rich repeat (NLR) receptors are activated by specific pathogen effectors. The *Arabidopsis* *TIR* (*Toll-Interleukin-1 receptor domain*)-*NLR* (denoted *TNL*) gene pair, *RPS4* and *RRS1*, confers resistance to *Pseudomonas syringae* pv *tomato* (*Pst*) strain DC3000 expressing the Type III-secreted effector, AvrRps4. Nuclear accumulation of AvrRps4, RPS4, and the TNL resistance regulator EDS1 is necessary for ETI. RRS1 possesses a C-terminal “WRKY” transcription factor DNA binding domain suggesting that important RPS4/RRS1 recognition and/or resistance signaling events occur at the nuclear chromatin. In *Arabidopsis* accession Ws-0, the *RPS4^Ws^*/*RRS1^Ws^* allelic pair governs resistance to *Pst*/AvrRps4 accompanied by host programed cell death (pcd). In accession Col-0, *RPS4^Col^*/*RRS1^Col^* effectively limits *Pst*/AvrRps4 growth without pcd. Constitutive expression of HA-StrepII tagged *RPS4^Col^* (in a *35S:RPS4-HS* line) confers temperature-conditioned *EDS1*-dependent auto-immunity. Here we show that a high (28°C, non-permissive) to moderate (19°C, permissive) temperature shift of *35S:RPS4-HS* plants can be used to follow defense-related transcriptional dynamics without a pathogen effector trigger. By comparing responses of *35S:RPS4-HS* with *35S:RPS4-HS* *rrs1-11* and *35S:RPS4-HS* *eds1-2* mutants, we establish that *RPS4^Col^* auto-immunity depends entirely on *EDS1* and partially on *RRS1^Col^*. Examination of gene expression microarray data over 24 h after temperature shift reveals a mainly quantitative *RRS1^Col^* contribution to up- or down-regulation of a small subset of *RPS4^Col^*-reprogramed, *EDS1*-dependent genes. We find significant over-representation of WRKY transcription factor binding W-box cis-elements within the promoters of these genes. Our data show that RRS1^Col^ contributes to temperature-conditioned RPS4^Col^ auto-immunity and are consistent with activated RPS4^Col^ engaging RRS1^Col^ for resistance signaling.

## INTRODUCTION

A critical layer of plant innate immunity is conferred by intracellular nucleotide binding-leucine rich repeat (NLR) receptors that guard against disease-promoting activities of pathogen effectors during infection (Dodds and Rathjen, 2010). Genes encoding NLR proteins represent the most diverse gene family in plants, probably as a result of pathogen selection pressure (Meyers et al., 2003; Yue et al., 2012). NLR receptors behave as ATP-driven molecular switches which become activated directly by physical association with an effector or indirectly through effector perturbations of a receptor-guarded co-factor (Maekawa et al., 2011; Bernoux et al., 2011a). Receptor activation triggers a robust anti-microbial response which is often accompanied by localized host programed cell death (pcd), although pathogen resistance can be uncoupled from pcd (Maekawa et al., 2011; Heidrich et al., 2012).

The NLR receptor family is broadly divided into two sub-classes based on different N-terminal putative signaling domains containing either Toll-Interleukin-1 receptor (TIR) homology, or a coiled-coil (CC) or other features, referred to, respectively, as TNLs and CNLs (Maekawa et al., 2011; Bernoux et al., 2011a). TNL and CNL receptor types signal in different ways for resistance (Wiermer et al., 2005; Venugopal et al., 2009). However, they all converge on the transcriptional machinery to amplify gene expression programs which operate in basal resistance against virulent (non-recognized) pathogens (Tao et al., 2003; Bartsch et al., 2006). Only a handful of TNL and CNL receptors have been characterized and many questions remain about where and how NLR are activated inside cells and the sequence of downstream signaling events leading to disease resistance. A number of functional NLR representatives from both sub-classes are nucleo-cytoplasmic and there is compelling evidence that NLR nucleo-cytoplasmic partitioning is important for full triggering of an immune response (Heidrich et al., 2012). Moreover, the *Arabidopsis* TNL protein SNC1 (Zhu et al., 2010b), tobacco TNL receptor N (Padmanabhan et al., 2013) and barley CNL receptor MLA1 (Chang et al., 2013) interact with transcription factors, suggesting a short route to the transcriptional machinery.

All functionally characterized TNL receptors depend on the nucleo-cytoplasmic immune regulator EDS1 (enhanced disease sensitivity1) for triggering resistance and pcd (Wiermer et al., 2005) and associations between several TNLs and EDS1 have been detected in *Arabidopsis* and tobacco, suggesting that EDS1 is part of an immune receptor signaling complex (Bhattacharjee et al., 2011; Heidrich et al., 2011; Kim et al., 2012). EDS1, in direct association with its signaling partner PAD4 (phytoalexin deficient4), is essential for basal resistance against virulent pathogens, measured as a slowing of pathogen growth without obvious TNL recognition or pcd (Jirage et al., 1999; Feys et al., 2001; Rietz et al., 2011). Based on interactions detected between EDS1 and *Pseudomonas syringae* Type III-secreted effectors AvrRps4 and HopA1, it was proposed that TNL receptors might guard the EDS1–PAD4 basal resistance machinery against interference by pathogen effectors as well as co-opting EDS1 as an early signaling component for execution of effector-triggered immunity (ETI; Bhattacharjee et al., 2011; Heidrich et al., 2011).

We are studying ETI in *Arabidopsis* mediated by the TNL receptor gene pair, *RPS4* and *RRS1*, in recognition of AvrRps4 derived from leaf-infecting *P. syringae* pv *pisi* (Hinsch and Staskawicz, 1996; Gassmann et al., 1999; Birker et al., 2009; Narusaka et al., 2009). Particular allelic forms of the same *RPS4 RRS1* pair also recognize an unrelated YopJ family effector, PopP2, secreted by root-infecting *Ralstonia solanacearum* bacteria (Deslandes et al., 2003; Narusaka et al., 2009). RPS4 accumulates as a nucleo-cytoplasmic protein associating with endo-membranes (Wirthmueller et al., 2007; Bhattacharjee et al., 2011). Notably, RPS4 nuclear accumulation conferred by a C-terminal NLS is essential for resistance to *P. syringae* pv *tomato* (*Pst*) expressing AvrRps4 (*Pst*/AvrRps4), although *RPS4* nucleo-cytoplasmic partitioning does not rely on the presence of either AvrRps4 or EDS1 (Wirthmueller et al., 2007; Heidrich et al., 2011). RRS1 is an atypical TNL in that it also possesses a C-terminal “WRKY” transcription factor DNA binding domain (Deslandes et al., 2002) known to recognize W-box consensus sequences within the promoters of defense-related genes (Rushton et al., 2010; Chen et al., 2013; Logemann et al., 2013). Analysis of the auto-immune phenotype of an *rrs1* (*slh1*) single amino acid insertion mutation in the WRKY domain abolishing DNA binding *in vitro*, led to the idea that *RRS1* exists as an auto-inhibited form at the chromatin in healthy tissues (Noutoshi et al., 2005). An effector trigger might then cause an RRS1 conformational switch to initiate resistance signaling. Other studies established that RRS1 interacts with *R. solanacearum* effector PopP2 (Deslandes et al., 2003; Tasset et al., 2010). PopP2 has an auto-acetyltransferase activity and this enzymatic function, coupled with recognition by a resistant RRS1-R allelic form, appear to be necessary for triggering resistance (Tasset et al., 2010). By contrast, AvrRps4 has no known enzyme activity but is proteolytically cleaved inside plant cells to produce an 11 kDa α-helical CC C-terminal fragment which is essential for *RPS4*/*RRS1* recognition (Sohn et al., 2009,^2012^). While association between AvrRPS4 and EDS1 was reported based on fluorescence resonance energy transfer–fluorescence life-time imaging (FRET–FLIM) and co-immunoprecipitation assays in tobacco and *Arabidopsis* (Bhattacharjee et al., 2011; Heidrich et al., 2011), another study argued against AvrRps4–EDS1 association based on negative interaction data (Sohn et al., 2012). Clearly, much needs to be resolved about the configurations of receptor pre-activation and signaling complexes and their precise relationship with the transcriptional machinery.

Resistance conditioned by TNL receptors is acutely sensitive to temperature with higher temperatures suppressing activated immune responses (Yang and Hua, 2004; Wang et al., 2009; Kim et al., 2010; Zhu et al., 2010a; Alcazar and Parker, 2011). Previously, we described an HA-StrepII epitope tagged RPS4 over-expression line (*35S:RPS4-HS*) in *Arabidopsis* accession Columbia (Col-0) which displays *EDS1*-dependent auto-immunity and stunting at 22°C, consistent with EDS1 being recruited coincidently or immediately downstream of activated RPS4 (Wirthmueller et al., 2007; Heidrich et al., 2011). Here we establish that auto-immunity in the *35S:RPS4-HS* plants grown at 22°C or shifted from a suppressive (28°C) to permissive (19°C) temperature depends fully on *EDS1* and partially on *RRS1^Col^*. We have used the 28–19°C temperature shift to induce RPS4^Col^ immunity and examine transcriptional reprograming in leaf tissues. This reveals a mainly quantitative contribution of RRS1^Col^ to up- and down-regulation of a discrete set of EDS1-dependent genes. The data suggest that RRS1 acts positively and at an early stage of RPS4 auto-immunity.

## MATERIALS AND METHODS

### PLANT MATERIALS AND GROWTH CONDITIONS

All mutant and transgenic lines used were in *Arabidopsis* accessions Columbia (Col-0) or Wassilewskija (Ws-0). Col *eds1-2* (Bartsch et al., 2006), *rps4-2* (Wirthmueller et al., 2007), *rrs1-11* (Birker et al., 2009), Ws *eds1-1* (Parker et al., 1996), *rps4-21*, *rrs1-1*, and *rps4-21/rrs1-1* (Narusaka et al., 2009) mutant lines, *35S:RPS4-HS* and *35S:RPS4-HS eds1-2* (Wirthmueller et al., 2007) have been described. The *35S:RPS4-HS rrs1-11* line was generated by crossing *35S:RPS4-HS* with *rrs1-11*. Plants were grown in soil in chambers under a 10/14 h day/night cycle (150–200 μE/m^2^s) and ∼65% relative humidity at 19, 22, or 28°C.

### BACTERIAL STRAINS

Bacterial strains *Pst* strain DC3000 and *Pst* DC3000 expressing AvrRps4 (*Pst*/AvrRps4) were obtained from R. Innes (Indiana University, Bloomington, USA) and grown as described (Hinsch and Staskawicz, 1996). *Pst* strain DC3000 expressing AvrRps4-HA or the AvrRps4-HA-NLS and AvrRps4-HA-NES variants from a pEDV6 vector, or a non-pathogenic *Pseudomonas fluorescens* (*Pfo*) strain for delivery of Type III-secreted effectors (Thomas et al., 2009) expressing AvrRps4-HA in pEDV6, have been described (Heidrich et al., 2011).

### BACTERIAL GROWTH ASSAYS

**FIGURE 1:**
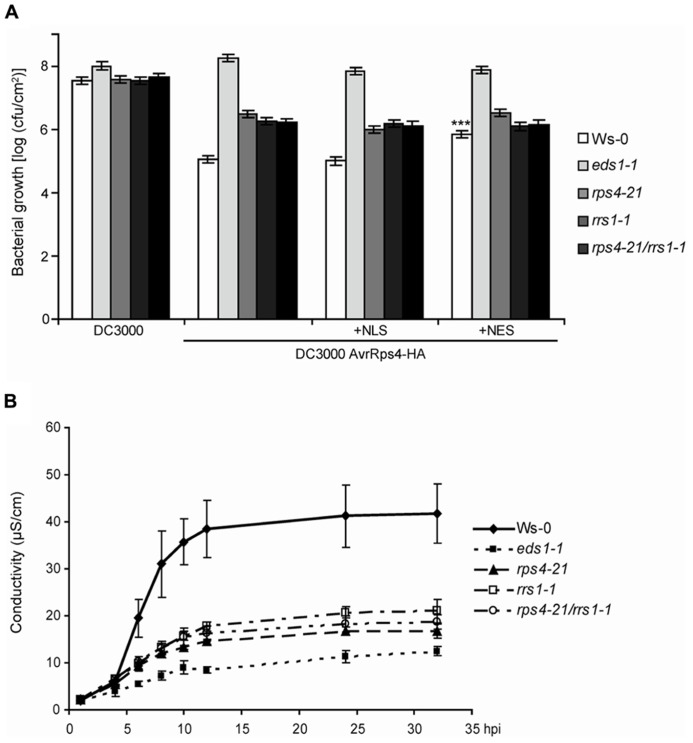
***RPS4^Ws^* and *RRS1^Ws^*** act cooperatively in AvrRps4-triggered bacterial resistance and pcd. **(A)** Four-week-old plants were spray-inoculated with virulent *Pst* DC3000 or *Pst* expressing AvrRps4-HA, AvrRps4-HA-NLS, or AvrRps4-HA-NES variants. Bacterial titers at 3 dpi are shown. All bacterial strains had similar entry rates measured at 3 hpi (data not shown). Replicate values were combined from three independent experiments with similar results and SEs calculated using a linear model. ***Significant difference (*p* < 0.001). **(B)** Ion leakage measurements were recorded at the indicated time points in leaf disks of 4-week-old Ws-0, *eds1-1*, *rps4-21*, *rrs1-1*, and *rps4-21 rrs1-1* plants after infiltration with *Pfo*-expressing AvrRps4-HA. Error bars represent standard errors of four samples per genotype. The experiment was performed three times with similar results.

For *Pst* spray infections, bacteria were adjusted to 1 × 10^8^ cfu/ml in 10 mM MgCl_2_ containing 0.04 % (v/v) Silwet L-77 (Lehle seeds, USA). *In planta* bacterial titers were determined 3 h after spray-infection (day 0) and 3 days post-infection (dpi) by shaking leaf disks in 10 mM MgCl_2_ with 0.01% Silwet L-77 at 28°C for 1 h, as described (Tornero and Dangl, 2001; Garcia et al., 2010). Infected plants were kept in a growth cabinet with a 10/14 h day/night cycle at 23°C. Mean values and standard errors (SEs) were calculated from at least three biological replicates per experiment. In the bacterial growth assays shown in **Figure 1A**, raw data was log_10_ transformed and all replicate values from three independent experiments analyzed using a linear model.

### ION LEAKAGE ASSAYS

For conductivity measurements after *Pfo* infiltration, leaves of 4-week-old-plants were infiltrated with 1.5 × 10^8^ cfu/ml bacteria in 10 mM MgCl_2_. Leaf disks were collected using a cork borer (6 mm diameter), floated in water for 30 min, and three leaf disks per measurement were subsequently transferred to a microtiter plate containing 3 ml distilled water. Conductivity of the solution was determined with a Horiba Twin B-173 conductivity meter at the indicated time points. Mean values and SE were calculated from four replicate measurements per genotype or bacterial strain. Experiments were repeated at least three times.

### PROTEIN IMMUNOBLOTTING

Total protein extracts from *Arabidopsis* leaves were prepared as previously described (Garcia et al., 2010). Protein concentrations were quantified and separated by sodium dodecyl sulfate-polyacrylamide gel electrophoresis (SDS-PAGE). Proteins were electro-blotted onto nitrocellulose membranes. Equal protein transfer was monitored by staining membranes with Ponceau S (Sigma-Aldrich). Membranes were blocked in a 5%-milk Tris buffer saline-Tween (TBST 20) solution before incubation in a 2% milk-TBST solution containing primary α-HA antibody (3F10; Roche) overnight. The appropriate horseradish peroxidase-conjugated secondary antibody (Santa Cruz Biotechnology) was applied and proteins were detected using enhanced chemiluminiscence reagent (ECL; Pierce Thermo Scientific).

### RT-PCR ANALYSIS OF DEFENSE GENE EXPRESSION

Total RNA was extracted from leaf material of 3-week-old plants using TriReagent (Sigma-Aldrich) according to the manufacturer’s protocol. A 1.5 μg of total RNA was incubated with 10 units of RNAse-free DNAse I (Roche) at 37°C for 30 min followed by heat-inactivation of the enzyme at 75°C for 10 min. Reverse transcription was performed with SuperScript II enzyme (Invitrogen) according to the manufacturer’s protocol. The following primer combinations were used for semi-quantitative real-time polymerase chain reaction (RT-PCR). Actin: fw GGCGATGAAGCTCAATCCAAACG, Actin: rev GGTCACGACCAGCAAGATCAAGACG; EDS1: fw TCATACGCAATCCAAATGTTTAC, EDS1: rev AAAAACCTCTCTTGCTCGATCAC; PBS3: fw CAACTTGTTAGAGGAGATCATCACACCC, PBS3: rev CCAGAAGGAGTCATGGATTCTTGTTTA; At5g26920: fw CGGAACAGCCCTAGTTTTCATGGG, At5g26920: rev GAGAAGACGAGAACGGTCCCGTACT; At5g27420: fw CTACTATTATCCGTGTCGGC, At5g27420: rev CGCGTCTAACCCACG.

### GENE EXPRESSION MICROARRAY ANALYSIS

Total RNA was prepared from 3.5-week-old plants grown at 28°C and shifted to 19°C for 0, 2, 8, and 24 h, using a QIAGEN Plant RNeasy kit. RNA quality was assessed on a Bioanalyzer (Agilent). Biotinylated cRNA was prepared and hybridized on Affymetrix ATH1-121501 “GeneChip” arrays, as described (Hajheidari et al., 2012). Briefly, biotinylated cRNA was made from 1 μg total RNA using the MessageAmp II-Biotin Enhanced Kit (Ambion). After amplification and fragmentation, 12.5 μg of cRNA were hybridized for 16 h at 45°C. Arrays were subsequently washed and stained in the Affymetrix Fluidics Station 450 using Fluidics Script FS450-004, and scanned with a GeneChip Scanner 3000 7G. For each condition, three Affymetrix ATH1 microarrays were hybridized with independent biological samples. Raw data for gene expression signals was extracted using the Affymetrix GeneChip Operating Software (version 1.4). For further data collection and assessment, R language version 2.15 (bioconductor project) was used. Probe signal values were subjected to GeneChip-robust multiarray average algorithm (GC-RMA; Wu and Irizarry, 2004). Probes which were below the background signal in all samples were not considered for further analysis. The results were analyzed by the following linear model using the lmFit function in the limma package in the *R* environment: *S*_gyr_ = *GY*_gyt_ + *R*_r_ + ε_gyr_, where *S* is log_2_ expression value, *GY*, genotype:time interaction, and random factors; *R* is biological replicate; ε, residual. The eBayes function in the limma package was used for variance shrinkage in calculating the *p*-values and the Storey’s *q*-values were calculated using the *q*-value function in the *q*-value package from the *p*-values (Storey and Tibshirani, 2003). Genes whose expression changes were *RRS1*-dependent upon temperature shift at any time point (*q*-values < 0.01 and >2-fold change) were selected (250 genes) for the clustering analysis. Heatmaps were generated by CLUSTER using uncentered Pearson correlation and complete linkage and were visualized by TREEVIEW (Eisen et al., 1998). Promoter sequences of the 250 *RRS1*-dependent genes were retrieved from the TAIR website^1^ with fixed 1000 bp sequences upstream of the translational start site. Over representation of the core W-box (TTGACY) was assessed using the promoter bootstrapping (POBO) application^2^ (Kankainen and Holm, 2004). One thousand pseudo-clusters of 250 genes were generated from the *RRS1*-dependent genes (Cluster2), all induced/suppressed genes upon temperature shift in Col (*q*-values < 0.01 and >2-fold change; Cluster 3), and the *Arabidopsis* genomic background (background). Statistical significance of the *t*-values generated by POBO was calculated using the linked Graphpad application for a two-tailed comparison: *Comparison of Cluster 2 and background (*p* < 0.0001); *Comparison of Cluster 2 and Cluster 3 (*p* < 0.0001); *Comparison of Cluster 3 and background (*p* < 0.0001). Analysis of gene ontology (GO) terms for the 250 *RRS1*-dependent genes was performed using Agrico^3^. Microarray data have been submitted to the Gene Expression Omnibus database (GEO accession no. GSE50019).

## RESULTS

### ANALYSIS OF *RPS4^WS^* AND *RRS1^WS^* COOPERATIVITY IN AVRRPS4-TRIGGERED RESISTANCE AND HR

In *Arabidopsis* accession Ws-0, resistance to *Pst* strain DC3000 expressing AvrRps4 (*Pst*/AvrRps4) after bacterial infiltration of leaves relies on genetic cooperation between *RPS4^Ws^* and *RRS1^Ws^* (Narusaka et al., 2009). We tested whether the *RPS4^Ws^* *RRS1^Ws^* dual resistance system also operates against spray-inoculated *Pst/*AvrRps4 which enter leaves through stomata. Suspensions of *Pst/*AvrRps4 were sprayed onto wild-type Ws-0, Ws *eds1-1*, the single Ws *rps4-21* and *rrs1-1* T-DNA insertion mutants or the *rps4-21* *rrs1-1* double-mutant (Narusaka et al., 2009), and bacterial growth measured in leaves. At 3 h post-inoculation, titers of all bacterial strains were similar (∼5 × 10^3^ cfu/cm^2^). At 3 days post-inoculation (dpi), the *rps4-21* *rrs1-1* double-mutant line displayed the same level of intermediate resistance as each *rps4-21* and *rrs1-1* single mutant, lying between fully resistant Ws-0 and fully susceptible *eds1-1* plants (**Figure 1A**). Therefore, *RPS4^Ws^* and *RRS1^Ws^* dual resistance to *Pst*/AvrRps4 also operates after bacterial infection through leaf stomata. Residual *EDS1*-dependent resistance in *rps4-21* *rrs1-1* to *Pst*/AvrRps4 infection (**Figure 1A**) is conferred by an *RPS4-* and *RRS1*-independent mechanism operating in Ws-0 and likely also in accession Col-0 expressing the respective *RPS4^Col^* and *RRS1^Col^* allelic variants (Birker et al., 2009; Sohn et al., 2012). We showed previously that resistance in Ws-0 and Col-0 to *Pst*/AvrRps4 could be effectively triggered by an AvrRps4-HA-NLS form targeted to nuclei and that this also required RPS4^Col^ nuclear accumulation (Heidrich et al., 2011). By contrast, enhanced nuclear export of AvrRps4-HA fused to a nuclear export sequence (AvrRps4-HA-NES) triggered low resistance but was able to trigger some pcd. Spray-inoculation of *Pst*-delivered AvrRps4-HA-NLS or AvrRps4-HA-NES variants (Heidrich et al., 2011) did not alter the partial resistance phenotype of the *rps4-21* and *rrs1-1* single or *rps4-21* *rrs1-1* double mutant lines (**Figure 1A**). Therefore, forced AvrRps4 localization to the nucleus or the cytoplasm does not alleviate the requirement for *RPS4^Ws^* or *RRS1^Ws^* in limiting bacterial infection or the extent of residual *RPS4* and *RRS1*-independent resistance.

Delivery of AvrRps4 from a non-infectious *Pfo* strain infiltrated into Ws-0 leaves triggers a strong macroscopic hypersensitive response (HR) which is abolished in Ws *eds1-1* mutant plants and reduced in *rps4-21* or *rrs1-1* mutants (Heidrich et al., 2011; Sohn et al., 2012). Resistance to *Pst*/AvrRps4 growth in *Arabidopsis* accession Col-0 is somewhat higher than in Ws-0 and depends on both the *RPS4^Col^* and *RRS1^Col^* allelic forms (Birker et al., 2009) but is accompanied by an extremely weak HR to *Pfo*/AvrRps4 (Heidrich et al., 2011; Sohn et al., 2012). Sohn et al. (2012) further showed that Col-0 transformed with a FLAG-tagged *RRS1^Ws^* transgene reconstituted a strong HR to infiltrated *Pfo*/AvrRps4, suggesting that *RRS1^Ws^* is a major determinant of AvrRps4-triggered pcd in Ws-0 or is able to boost the existing *RPS4^Col^*/RRS1^Col^ low-level pcd response. We performed a quantitative ion leakage assay over 36 h in leaves of Ws-0, the *rps4-21* and *rrs1-1* single mutants, and *rps4-21* *rrs1-1* double mutants after leaf infiltration of *Pfo*/AvrRps4. Ws *eds1-1* mutant leaves were infiltrated alongside as a non-responding control. As shown previously (Heidrich et al., 2011), Ws-0 leaves produced a rapid HR reaching a peak at 12–16 h after infiltration, whereas *eds1-1* leaves produced base line conductivity of ∼10 μS/cm over the ion leakage time course (**Figure 1B**). Responses of the single and double *rps4-21* *rrs1-1* mutants were all intermediate between Ws-0 and *eds1-1* (**Figure 1B**). Therefore, there is genetic cooperativity between *RPS4^Ws^* and *RRS1^Ws^* in eliciting host pcd and in partially restricting to *Pst*/AvrRps4 bacterial growth.

### *RRS1^Col^* CONTRIBUTES TO AUTO-ACTIVATED *RPS4^Col^* PLANT STUNTING AND IMMUNITY

**FIGURE 2:**
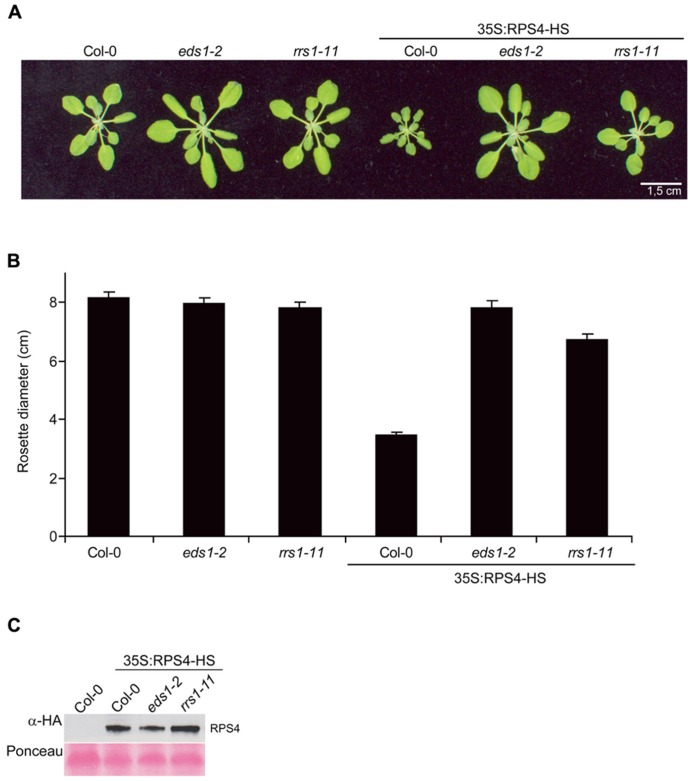
**Mutation of *RRS1^Col^* partially suppresses *35S:RPS4-HS* stunting. (A)** Growth at 22°C of representative 3.5-week-old Col-0, *eds1-2*, and *rrs1-11* and the same backgrounds containing the *35S:RPS4-HS* transgene. Scale bar, 1.5 cm. **(B)** Quantification of rosette diameters at 3.5 weeks of lines shown in **(A)**. **(C)** Immunoblot analysis of total leaf protein extracts separated by SDS-polyacrylamide gel electrophoresis (SDS-PAGE) from the 3.5-week-old *35S:RPS4-HS* transgenic leaf tissues in Col-0, *eds1-2*, and *rrs1-11* backgrounds, probed with α-HA antibody. Ponceau S staining shows equal transfer of protein samples to the membrane. Two independent experiments gave similar results.

We reported that a Col-0 line constitutively expressing functional HA-StrepII-tagged genomic *RPS4^Col^* under control of the CaMV *35S* promoter (referred to here as *35S:RPS4-HS*) exhibits *EDS1*-dependent auto-immunity and stunting at 22°C (Wirthmueller et al., 2007; Heidrich et al., 2011). Given the tight functional relationship between the *RPS4^Ws^* and *RRS1^Ws^* allelic pairs in accession Ws-0, and presumably between *RPS4^Col^* and *RRS1^Col^* in Col-0 for resistance to *Pst*/AvrRps4, we investigated whether *RRS1^Col^* also has a role in *35S:RPS4-HS*-triggered auto-immunity. A Col *rrs1* null mutant allele (*rrs1-11*; Birker et al., 2009) was crossed into the *35S:RPS4-HS* background and a line selected that was homozygous for the *35S:RPS4-HS* transgene and *rrs1-11*. The same *35S:RPS4-HS* line crossed into a Col *eds1-2* null mutant was used as a control with suppressed *RPS4* auto-immunity. As anticipated, *35S:RPS4-HS* plants were severely stunted after 3–4 weeks growth and *35S:RPS4-HS* *eds1-2* plants exhibited no growth inhibition at 22°C (**Figures 2A,B**). Steady-state RPS4-HS protein accumulation in *35S:RPS4-HS* *eds1-2* was slightly reduced compared to the *35S:RPS4-HS* line (**Figure 2C**). Mutation of *RRS1^Col^* caused intermediate *35S:RPS4-HS* stunting at 22°C (**Figures 2A,B**) but did not affect RPS4-HS accumulation (**Figure 2C**). Therefore, *RRS1^Col^* contributes positively to *RPS4^Col^* auto-immunity at the level of plant growth inhibition. We concluded that the RRS1^Col^ protein likely plays a role in resistance signaling triggered by an auto-activated RPS4^Col^ receptor, besides its presumed role in AvrRps4 recognition (Birker et al., 2009; Narusaka et al., 2009).

**FIGURE 3:**
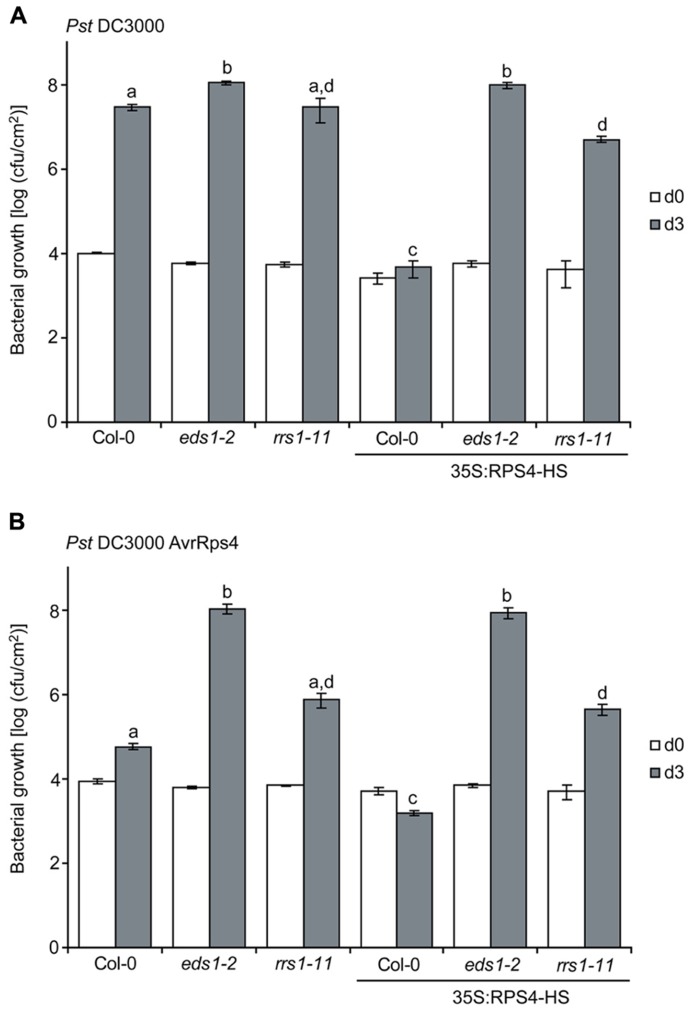
***RRS1^Col^* contributes to enhanced basal and AvrRps4-triggered resistance of *35S:RPS4-HS* at 22°C.** 3.5-week-old plants of the indicated lines grown at 22°C were spray-inoculated with virulent *Pst* DC3000 **(A)** or avirulent *Pst*/AvrRps4 **(B)** bacteria in the same experiment. Bacterial titers were measured at 3 hpi (d0) indicating bacterial entry rates and at 3 dpi (d3). Standard errors were calculated from three biological samples per genotype. Letters (a,b,c,d) indicate significant differences (*p* < 0.05) calculated by a Student’s *t*-test. Experiments were performed independently three times with similar results.

We then tested whether *35S:RPS4-HS* plants grown at 22°C display enhanced basal resistance to virulent *Pst* strain DC3000 and the influence of *rrs1-11* compared to *eds1-2* on the *35S:RPS4-HS* basal resistance phenotype. Col-0 wild-type, *eds1-2*, and *rrs1-11* plants were grown alongside *35S:RPS4-HS*, *35S:RPS4-HS* *eds1-2*, and *35S:RPS4-HS* *rrs1-11* plants for 3.5 weeks at 22°C and then spray-inoculated with *Pst* DC3000 for bacterial growth assays. The *rrs1-11* mutant supported similar *Pst* DC3000 growth as Col-0 wild type (**Figure 3A**) and therefore did not exhibit an enhanced disease susceptibility phenotype (which would be indicative of a loss of basal resistance), in contrast to *eds1-2* (**Figure 3A**). The *35S:RPS4-HS* plants exhibited strongly enhanced basal resistance to *Pst* DC3000 which was abolished by *eds1-2* and partially suppressed by *rrs1-11* (**Figure 3A**). We concluded that auto-immunity exhibited by *35S:RPS4-HS* at 22°C involves *RRS1^Col^* for enhancing *EDS1*-dependent basal resistance responses.

We spray-inoculated the same set of plants with *Pst*/AvrRps4 and found that the high basal resistance in *35S:RPS4-HS* (see **Figure 3A**) was slightly increased by AvrRps4 and was also fully *EDS1*-dependent (**Figure 3B**). The *35S:RPS4-HS* *rrs1-11* plants displayed intermediate loss of resistance to *Pst*/AvrRps4 (**Figure 3B**), suggesting that an *RPS4^Col^* RRS1^Col^-independent mechanism also plays a role in *35S:RPS4-HS* immunity to *Pst*/AvrRps4. The results show that *RRS1^Col^* contributes to *RPS4^Col^* auto-immunity. In genetically recruiting *EDS1* and *RRS1^Col^*, while retaining an *RRS1^Col^*-independent resistance component (**Figure 3B**), we reasoned that the *35S:RPS4-HS* auto-activated immune system might be useful for measuring *RPS4*/*RRS1*-triggered defense pathway transcription dynamics without needing to infect with the pathogen.

### A HIGH TO LOW TEMPERATURE SHIFT INDUCES *35S:RPS4-HS* AUTO-IMMUNITY

In *Arabidopsis*, suppression of basal and effector-triggered TNL immunity at high temperature (>25°C) is associated with lowered expression of defense pathway genes, including *EDS1*, and reduced feed-forward defense amplification (Yang and Hua, 2004; Wang et al., 2009). We therefore investigated whether shifting plants from high temperature (28°C, non-permissive for *Arabidopsis* TNL resistance) to a lower temperature (19–22°C, permissive for TNL resistance) could be used to turn on *RPS4* auto-immunity synchronously in leaf tissues.

**FIGURE 4:**
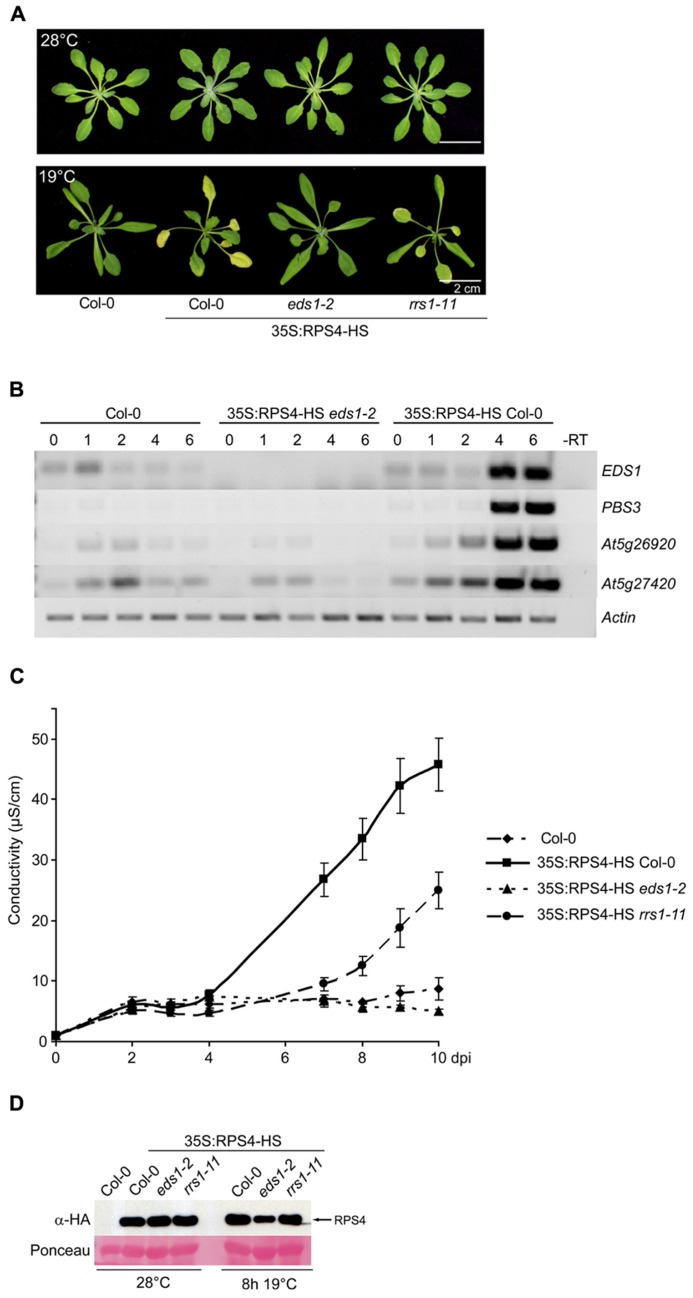
**A 28 to 19°C temperature shift induces *RPS4-HS* auto-immunity. (A)** Growth of 3.5-week-old *35S:RPS4-HS* plants at 28°C (upper panel) and 6 days after moving to 19°C (lower panel). Scale bars, 2 cm. **(B)** Semi-quantitative RT-PCR of known *Pst*/AvrRps4-responsive, *EDS1*-dependent genes over 0–6 h after temperature shift of Col-0, *35S:RPS4-HS* *eds1-2*, and *35S:RPS4-HS* Col-0 plants, as indicated. **(C)** Ion leakage measurements made over a 10-day period after shift from high to low temperature (dps) in leaf disks of the different 3.5-week-old *35S:RPS4-HS* lines and Col-0 wild-type, as indicated. Error bars represent standard errors of four samples per genotype. Three independent experiments gave similar results. **(D)** Immunoblot analysis of total leaf protein extracts from 3.5-week-old *35S:RPS4-HS* lines grown at 28°C and shifted to 19°C for 8 h, separated by SDS-polyacrylamide gel electrophoresis (SDS-PAGE) and probed with α-HA antibody. Ponceau S staining shows equal transfer of protein samples to the membrane.

The *35S:RPS4-HS* plants grew similarly to wild type Col-0 at 28°C (**Figure 4A**) and showed no constitutive defense gene expression (**Figure 4B**). Moving *35S:RPS4-HS* plants from 28 to 19°C induced expression of *EDS1* itself and several known *Pst*/AvrRps4-responsive, *EDS1*-dependent defense-related genes (Bartsch et al., 2006) at 4 and 6 h post-temperature shift (hps; **Figure 4B**). Col-0 wild type and *35S:RPS4-HS* *eds1-2* plants subjected to the same temperature change did not show induction of these genes at 4 and 6 hps (**Figure 4B**). In multiple repeats, the 28 to 19°C temperature shift proved to be an easy and highly reproducible *EDS1*-requiring defense gene inductive switch for *35S:RPS4-HS* plants.

Macroscopic symptoms of auto-immunity were first seen as leaf chlorosis in *35S:RPS4-HS* plants, starting at 3–4 days after the 28 to 19°C temperature shift and showing complete *EDS1*-dependence (**Figure 4A**). In conductivity assays for cell death, ion leakage from *35S:RPS4-HS* leaf disks started to rise significantly between 4 and 6 days post-shift (dps) but did not increase in *35S:RPS4-HS* *eds1-2* or wild-type Col-0 (**Figure 4C**). We tested the *35S:RPS4-HS* *rrs1-11* line under the same conditions and found that progression of leaf chlorosis (**Figure 4A**) and ion leakage (**Figure 4C**) was intermediate between *35S:RPS4-HS* and *35S:RPS4-HS* *eds1-2* plants. Steady-state RPS4-HS protein accumulation was not strongly affected by temperature or the *rrs1-11* mutation, but was slightly lower in *eds1-2* at 8 h after temperature shift (**Figure 4D**). Collectively, these data show that *RRS1^Col^* contributes to temperature-conditioned *35S:RPS4-HS* auto-immunity at the level of leaf chlorosis and pcd.

### *RRS1^Col^* SUPPORTS TRANSCRIPTIONAL REPROGRAMING OF A DISCRETE SET OF *EDS1*-DEPENDENT GENES IN TEMPERATURE-SHIFTED *35:RPS4-HS* PLANTS

**FIGURE 5:**
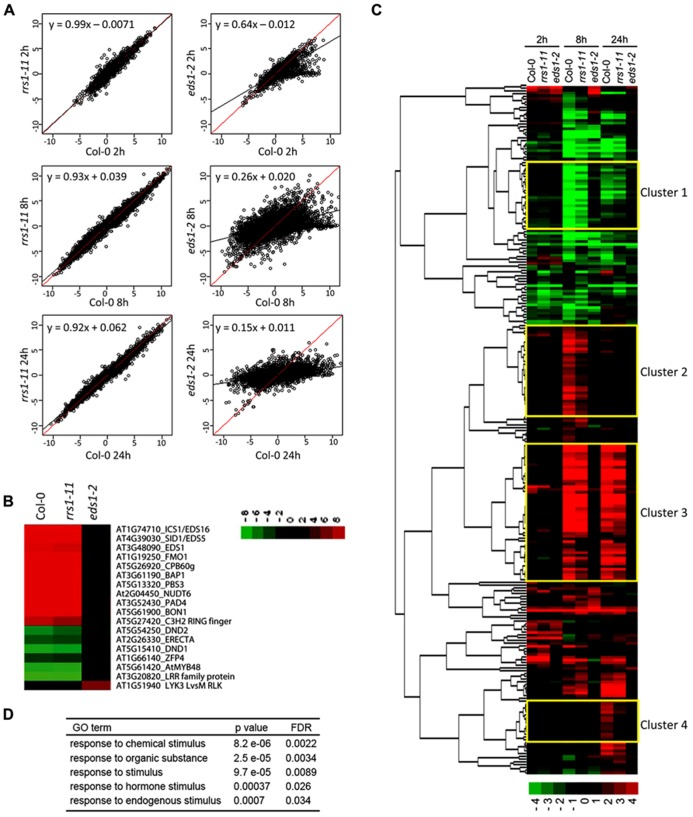
***RRS1^Col^* contributes to *EDS1*-dependent gene expression changes in *RPS4^Col^* auto-immunity.** Gene expression microarray analysis was performed on leaf RNAs of 3.5-week-old *35S:RPS4-HS* transgenic plants in the Col-0, *rrs1-11*, or *eds1-2* backgrounds at 0, 2, 8, and 24 h after temperature shift. **(A)** Induced or repressed genes (*q*-values < 0.01 and >2-fold changes) in *35S:RPS4-HS* Col-0 upon temperature shift at any time point and the log_2_ ratios compared to 0 h are plotted. Linear regression lines indicate log_2_ ratios in *35S:RPS4-HS* Col-0 (red) and the *35S:RPS4-HS* *rrs1-11* or *35S:RPS4-HS* *eds1-2* mutant lines (black). **(B)** Log_2_ gene expression ratios at 8 h after temperature shift compared to 0 h in *35S:RPS4-HS* Col-0 plants for previously identified *Pst*/AvrRps4-triggered *EDS1*-dependent genes, shown by Heatmap clustering analysis. **(C)** Heatmap clustering of 250 genes whose expression changes are *RRS1*-dependent in *35S:RPS4-HS* after temperature shift at any time point (*q*-values < 0.01 and >2-fold change). Expression patterns for the 250 genes in *35S:RPS4-HS* Col-0, *35S:RPS4-HS* *rrs1-11*, and *35S:RPS4-HS* *eds1-2* lines are shown at 2, 8, and 24 h. Highlighted clusters 1–4 are described in the text. **(D)** GO term analysis of the 250 *RRS1*-dependent genes.

In the above assays, we established that *35S:RPS4-HS* 28/19°C-shifted leaf tissues resemble *Pst*/AvrRps4-infected plants at 22°C with respect to complete *EDS1*- and partial *RRS1^Col^*-dependence in chlorotic and pcd phenotypes. However, the temperature shift will have physiological effects unrelated to immunity (Penfield, 2008; McClung and Davis, 2010). We therefore performed gene expression microarray analysis of *35S:RPS4-HS*, *35S:RPS4-HS* *rrs1-11*, and *35S:RPS4-HS* *eds1-2* leaf mRNAs at 0 h (28°C), 2, 8, and 24 hps to 19°C in order to determine the relative contributions of RRS1^Col^ and EDS1 to temperature-conditioned *35S:RPS4-HS* transcriptional reprograming. Profiling of polyA^+^ RNAs was performed using Affymetrix ATH1 GeneChips (see Materials and Methods). We first selected genes whose expression was significantly up- or down-regulated (*q*-values < 0.01 and >2-fold change) in *35S:RPS4-HS* over all time points compared to non-shifted *35S:RPS4-HS* plants at 28°C (t0; 10277 genes in total). Hence, there is extensive reprograming of transcription in *35S:RPS4-HS* leaves over 24 hps. We then compared the global gene expression profiles of *35S:RPS4-HS*, *35S:RPS4-HS* *rrs1-11*, and *35S:RPS4-HS* *eds1-2* at 0, 2, 8, and 24 hps by plotting changed transcripts in *35S:RPS4-HS* *rrs1-11* or *35S:RPS4-HS* *eds1-2* on a linear regression curve (red) against the regression curve set by *35S:RPS4-HS* transcript changes (black; **Figure 5A**). This analysis shows that expression changes in *35S:RPS4-HS* *rrs1-11* broadly resemble those of *35S:RPS4-HS* over the 24 h time course (**Figure 5A**). Therefore, loss of RRS1^Col^ function has little effect on RPS4-HS transcriptional reprograming overall. Many gene expression changes in *35S:RPS4-HS* at 2 hps (80%) were also similar in *35S:RPS4-HS* *eds1-2*, as seen by the near congruence of the red and black regression curves (**Figure 5A**). A measurable impact of *eds1-2* on expression changes in *35S:RPS4-HS* was observed at 8 and 24 hps, with most differences between the two lines established already at 8 hps (**Figure 5A**). These data show that EDS1 contributes substantially to RPS4-HS-triggered transcriptional reprograming following an early EDS1-independent phase that is likely due to the temperature shift *per se* and not directly related to *RPS4* auto-immunity. We then selected a sample of defense-related genes whose up- or down-regulation was established in a previous gene expression microarray study as *EDS1*- and *PAD4*-dependent at 6 h after leaf infiltration with *Pst*/AvrRps4 bacteria at 22°C (Bartsch et al., 2006; Zhu et al., 2010b). The pattern of AvrRps4-triggered induction or repression of the genes was recapitulated at 8 h post-temperature shift in *35S:RPS4-HS* and *35S:RPS4-HS* *rrs1-11* and was strongly *EDS1*-dependent, as shown in a heatmap (**Figure 5B**). This suggests that major defense-related transcriptional changes requiring *EDS1* in *Pst*/AvrRps4-infected tissues are qualitatively similar at 8 hps in the temperature-conditioned *RPS4* auto-immune response.

We investigated whether a subset of the total 10227 genes exhibiting changed expression over the *35S:RPS4-HS* temperature shift experiment was affected by *rrs1-11* by selecting genes whose up- or down-regulation showed dependence on *RRS1^Col^* for at least one time point (*q*-values < 0.01 and >2-fold change). Altogether, 250 genes fitted this pattern with most showing reduced up-regulation in *35S:RPS4-HS* *rrs1-11* tissues compared to *35S:RPS4-HS*. The 250 genes displayed partial *RRS1^Col^*- and strong *EDS1*-dependence for expression changes, as shown in the heatmap (**Figure 5C**). Hence, the effect of the *rrs1-11* mutation is mainly quantitative in the *35S:RPS4-HS* temperature-conditioned system. Analysis of GO terms enriched among the 250 genes shows a high representation of genes responsive to chemical, hormone, and other endogenous stimuli (**Figure 5D**). In a clustering analysis of the 250 “*RRS1^Col^*-dependent” genes (see Materials and Methods), four gene clusters were of interest (**Figure 5C**). In Cluster 1, genes are grouped that show *RRS1^Col^*-dependent repression at 8 and 24 h. Cluster 2 contains genes that are up-regulated at 8 hps and show an *RRS1^Col^* contribution to induction. Cluster 3 has genes up-regulated at 8 and 24 hps and showing *RRS1^Col^*-dependence at both time points. In Cluster 4, a discrete set of genes displaying *RRS1^Col^*-dependence in up-regulation at 24 hps is displayed. Interestingly, distinct sub-clusters of genes with strong *RRS1^Col^*-dependence are observed within Clusters 3 and 4 (**Figure 5C**). We concluded that *RRS1^Col^* has a measurable positive effect on expression of a subset of *EDS1*-dependent genes in *35S:RPS4-HS* auto-immunity.

**FIGURE 6:**
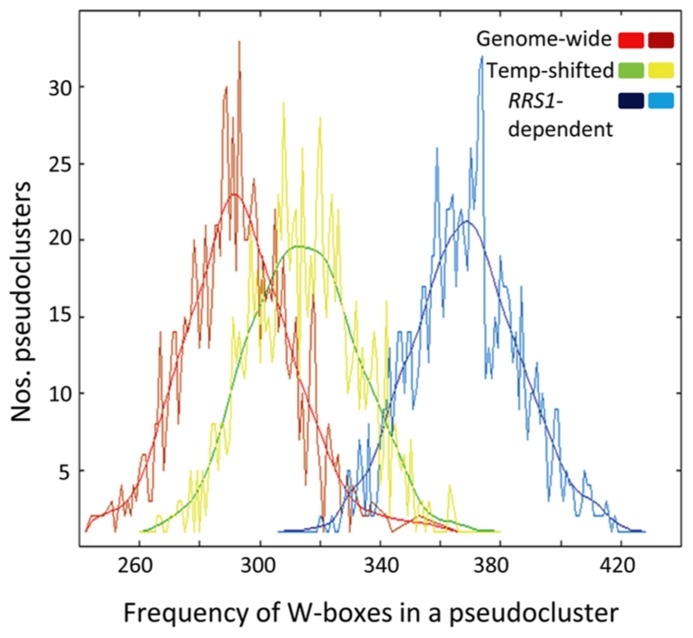
**W-boxes are highly enriched in promoters of *RRS1*-dependent genes.** POBO analysis of the motif distribution in 1000 bp promoters of *RRS1*-dependent genes. One thousand pseudo-clusters of the 250 *RRS1*-dependent genes, genes regulated by the temperature shift (Temp-shifted) and randomly selected genes from *35S:RPS4-HS* (Genome-wide) are shown. Jagged lines indicate motif frequencies from which a fitted curve was derived. The W-box (TTGACY) is significantly over-represented in promoters of the *RRS1*-dependent genes compared to temperature-responsive genes or genes from the genome background with *p*-values < 0.0001.

Because *RRS1^Col^* encodes a functional TNL receptor with a C-terminal “WRKY” transcription factor DNA-binding domain recognizing W-box elements, we investigated if W-box cis-elements are enriched in the promoters of the 250 *RRS1^Col^*-dependent genes. As shown in **Figure 6**, analysis of the core W-box motif (TTGACY) in promoters of these genes by POBO (Materials and Methods) shows that enrichment of this motif is highly significant (*p*-value < 0.0001) compared to randomly selected promoters from all *Arabidopsis* genes. Since the W-box is known to be enriched in promoters of genes that are responsive to biotic stresses (Rushton et al., 2010), we also compared W-box enrichment between promoters of the 250 *RRS1^Col^*-dependent genes and promoters from randomly selected *35S:RPS4-HS*-regulated genes. The POBO analysis showed that W-boxes remain significantly enriched (*p*-value < 0.0001) in the promoters of the *RRS1^Col^*-dependent genes (**Figure 6**). These results suggest that RRS1^Col^ acts on a subset of *35S:RPS4-HS* reprogramed genes directly or indirectly through the presence of W-box elements in their gene promoters.

## DISCUSSION

NLR receptors are usually activated upon specific pathogen effector recognition to trigger a timely and balanced innate immune response. In the absence of a corresponding effector, tight regulation of NLR receptors is enforced by restricting *NLR* gene expression and ensuring NLR associations with inhibitory co-factors (Heidrich et al., 2012; Staiger et al., 2013). Auto-immunity producing stunting and constitutive activation of resistance and cell death pathways can occur when NLRs are released from inhibition either by NLR over expression or loss-of-function mutations in negative factors (Heidrich et al., 2012; Staiger et al., 2013). An outstanding question is to what extent auto-activated NLR processes mirror those triggered by authentic effector recognition. For TNLs there is compelling evidence that auto-activated receptors connect immediately to a *bona fide* TNL resistance signaling pathway involving the basal resistance regulator EDS1 (Zhang et al., 2003; Yang and Hua, 2004; Wirthmueller et al., 2007; Huang et al., 2010). Detection of EDS1 in complexes with several NLRs (Bhattacharjee et al., 2011; Heidrich et al., 2011; Kim et al., 2012) is also consistent with EDS1 being an integral and early component of TNL resistance. Thus, effector- and auto-activated TNL signaling steps are likely to be related, although constitutive resistance clearly has deleterious pleiotropic effects on growth and development.

Here we provide evidence that *EDS1*-dependent auto-immunity in an *Arabidopsis* *RPS4^Col^* over-expression line (*35S:RPS4-HS*) has a partial requirement for *RRS1^Col^*, the genetic partner of *RPS4^Col^* in ETI (Birker et al., 2009; Narusaka et al., 2009). This partial dependence on *RRS1^Col^* is seen in plants grown at 22°C that exhibit constitutive basal resistance (**Figure 3**) and after shifting plants from high (28°C) to moderate (19°C) temperature to induce defense-related transcriptional reprograming, chlorosis, and pcd (**Figures 4 and 5**). Hence, RPS4 auto-immunity does not fully override a requirement for RRS1. Therefore, we reasoned that the dual RPS4–RRS1 resistance system might involve RPS4–RRS1 cooperation beyond initial effector recognition steps to include aspects of downstream resistance signaling. Alternatively, part of the RPS4 auto-activation mechanism involves processes that also occur during effector activation, such as particular NLR conformational transitions (Collier and Moffett, 2009; Lukasik and Takken, 2009). Reduced *RPS4^Col^* auto-immunity in *rrs1-11* mirrors the intermediate loss of resistance in *rrs1-11* mutants to *Pst*/AvRps4 bacteria (**Figures 1 and 3**). Therefore, it is possible that in both backgrounds an *RPS4*/*RRS1*-independent pathway contributes to the residual resistance (Birker et al., 2009; Sohn et al., 2012). Although the precise nature of effector- and auto-triggered RPS4–RRS1 activation events needs to be resolved, the fact that temperature-induced RPS4 immunity mirrors ETI in displaying complete dependence on EDS1 and partial dependence on RRS1 is significant. The temperature-conditioned RPS4 auto-immune system presents a potentially powerful tool to examine dynamic TNL signaling and transcriptional events in leaf tissues.

Pairing of *RPS4* and *RRS1* genes and their homologs in a head-to-head tandem arrangement is evolutionarily conserved, underscoring functional significance of the inverted TNL organization (Gassmann et al., 1999; Narusaka et al., 2009). *RRS1*, a representative of the TNL-A clade, exhibits higher sequence diversity among *Arabidopsis* accessions than *RPS4*, as a member of the TNL-B clade (Meyers et al., 2003; Narusaka et al., 2009). This, together with finding that the RRS1 interacts directly with the *R. solanacearum* effector PopP2 inside nuclei points to RRS1 as a direct effector recognition component, although interaction alone is not sufficient for triggering RRS1 resistance (Deslandes et al., 2003; Tasset et al., 2010). Noutoshi et al. (2005) proposed an attractive model for RRS1 “restraint” and activation based on analysis of an auto-activated *slh1* WRKY domain mutation. In the model, RRS1 in non-elicited cells resides at sites on the chromatin as an auto-inhibited NLR. Subsequent studies revealing *RRS1*–*RPS4* genetic cooperativity in resistance to AvrRps4 and PopP2, and an unknown *Colletotrichum higginsianum* effector (Birker et al., 2009; Narusaka et al., 2009), raised the prospect that effector recognition might be conferred by an auto-inhibited RPS4–RRS1 complex which becomes activated via RPS4–RRS1 conformational changes at the chromatin. Because our data indicate that RRS1 contributes to RPS4 auto-immunity, we propose that signaling events also involve RRS1 with RPS4, as well as EDS1, in what might be a “reconfigured” receptor complex, possibly mediated through TIR–TIR interactions (Mestre and Baulcombe, 2006; Bernoux et al., 2011b). The fact that neither *rrs1* nor *rps4* null mutant displays constitutive resistance also argues against resistance pathway activation simply being due to release of one or other component from an auto-inhibited complex. An interesting but complicating issue is that EDS1 was found to interact with the AvrRps4 effector in FRET–FLIM and co-immunoprecipitation studies, implying that EDS1 contributes to effector recognition as well as being an integral component of the TNL resistance pathways (Bhattacharjee et al., 2011; Heidrich et al., 2011). Notably, EDS1 interacts with two effectors, AvrRps4 and HopA1, recognized, respectively, by TNLs RPS4/RRS1 and RPS6 (Bhattacharjee et al., 2011; Heidrich et al., 2011). Thus, TNL pre- and post-activation events in these recognition systems might be closely intertwined.

Temperature-induced *RPS4* auto-immunity produces an exaggerated transcriptional response compared to ETI probably through an *EDS1*-dependent transcriptional feed-forward loop (Wang et al., 2009; Zhu et al., 2010a). At 2 h post-temperature shift, analysis of the gene expression microarray data revealed mainly *EDS1*-independent transcriptional reprograming of *35S:RPS4-HS* plants which we attribute to a “temperature” effect (**Figure 5A**). The small sector (20%) of *EDS1*-dependent changes at 2 h will be examined in a more detailed expression time series over 1–4 h to identify earliest *EDS1* and, potentially, *RRS1* effects. At 8 h after temperature shift, transcriptional reprograming was largely *EDS1*-dependent (**Figure 5**) and qualitatively similar to ETI for a panel of AvrRps4-triggered *EDS1*-dependent induced and repressed genes (**Figure 5B**). A quantitative contribution of *RRS1* was detected also at 8 and 24 h after temperature shift in 250 of the *EDS1*-dependent down and up-regulated genes (**Figure 5**). An auxiliary role of RRS1 in EDS1-mediated gene expression is reminiscent of the contribution of WRKY18 to NPR1-dependent basal defense responses (Wang et al., 2006) and might reflect a common feature of WRKY-containing transcriptional immune regulators. Notably, several sub-clusters within the *RRS1*-dependent genes display strong *RRS1*-dependence in expression at 8 or 24 h (**Figure 5C**; Clusters 3 and 4). Whether any of these genes are direct targets of RRS1 (or RPS4) is not known but the high representation of W-boxes in their promoter elements (**Figure 6**) suggests that WRKY-domain protein recruitment might be an important modulator of expression. Current evidence indicates that the dynamics of WRKY transcription factor binding of promoters are complex and likely to involve reconfigurations from repressive to inductive transcription complexes at the chromatin, as well as functional redundancy between WRKY transcription factors (Rushton et al., 2010; Chen et al., 2013; Logemann et al., 2013; Schon et al., 2013).

## CONCLUSION

Our data show that RRS1^Col^ positively contributes to RPS4^Col^ auto-immunity induced by a high to moderate temperature shift. The temperature-activated RPS4 over-expression system can help to illuminate the molecular role of RRS1 in this TNL resistance partnership and the hierarchy of defense-related transcriptional reprograming events.

## Conflict of Interest Statement

The authors declare that the research was conducted in the absence of any commercial or financial relationships that could be construed as a potential conflict of interest.

## Footnotes

1

2

3
